# Uncovering multiple influences on space use by deer mice using large ecological networks

**DOI:** 10.1007/s00442-025-05731-2

**Published:** 2025-06-04

**Authors:** Sean O’Fallon, Noa Pinter-Wollman, Karen E. Mabry

**Affiliations:** 1 Department of Ecology and Evolutionary Biology, University of California Los Angeles, 621 Charles E. Young Drive South, Los Angeles, CA 90095 USA; 2 Department of Biology, New Mexico State University, 1200 S Horseshoe St, Las Cruces, NM 88003 USA

**Keywords:** Behavioral ecology, Home range, NEON, *Peromyscus*, Space use, Utilization distribution

## Abstract

Space use by animals is affected by multiple factors; previous researchers have examined the effects of influences, such as sex, body condition, and population density on home range area. However, evaluating the simultaneous influences of multiple factors on animal space use has been relatively intractable due to sample size limitations. We capitalize on National Ecological Observatory Network (NEON) data to ask what factors determine space use by deer mice (genus *Peromyscus*). We examined data from 10 years of repeated captures of individually-identified mice at 36 sites across North America. We confirmed previous findings that males have larger home ranges than females and that home range area decreases with increasing animal density. In addition, our large sample size (*N* = 2,420 individuals) enabled us to examine the interacting influences of these, and other, phenotypic and extrinsic factors using a robust statistical framework. We found that the relationship between body condition and home range area differs between male and female mice, and that habitat type, latitude, and animal density all interact to influence space use. We conclude that data from large ecological networks can be used to examine important behavioral questions that have long eluded investigators.

**Supplementary Information:**

The online version contains supplementary material available at 10.1007/s00442-025-05731-2.

## Introduction

Space use by animals is influenced by multiple factors, both internal (phenotypic) and external (environmental) to an individual. For example, phenotypic effects on space use, such as sex and body condition, have been thoroughly investigated, as have socioecological conditions, such as population density, resource availability, and habitat variation (Börger et al. 2008; van Beest et al. 2011). Furthermore, the ways in which animals use space have important fitness consequences (Mcloughlin et al. 2007). Although previous work has predominantly examined how each factor affects space use separately, these factors are integrated and work in synergy (Ofstad et al. 2019; van Beest et al. 2011; Mcloughlin & Ferguson 2000). Sample size limitations have hampered integrative studies that examine the relative influences of multiple interacting effects on variation in space use simultaneously. With recent developments of large ecological datasets, new opportunities to examine the interactions among various factors on space use are opening up.

The concept of a home range, an area that contains all of the resources that an animal needs for daily life (Burt 1943), is often used to quantify space use by terrestrial animals. The internal (phenotypic) (Spencer et al. 1990) and external (environmental) (Börger et al. 2006a) factors that can influence home range area may reinforce or counteract each other. For example, an animal in good physical condition may be able to move over and utilize a larger area than an animal in poor condition. However, the effect of body condition on home range area may be offset by habitat quality: a more competitive animal that establishes its home range in a resource-rich environment can meet its daily needs in a smaller area. Indeed, interactions between body mass and habitat type have been observed in ungulates (Ofstad et al. 2016). Given the potentially conflicting influences of multiple factors on home range area, it is important to examine the simultaneous effects of these factors on animal space use. Yet, most studies of animal space use quantify home range area for one species in one location in relation to one or few factors that influence home range area, which makes it difficult to uncover synergies among factors that impact animal space use. Some researchers have dealt with the limitations of isolated home range studies through meta-analysis; for example, studies of metabolic scaling demonstrate that home range area scales with body size across species (Jetz et al. 2004; Kelt & Van Vuren 2001; Ofstad et al. 2016). Further meta-analyses should be facilitated by the *HomeRange* database of home range estimates for almost 1,000 mammal species (Broekman et al. 2023) such as the analysis of environmental drivers of home range size in both terrestrial and marine mammals (Broekman et al. 2024).

Despite advances in large-scale meta-analyses of influences on home range area across taxa, simultaneous examination of multiple phenotypic and external factors on space use in a single taxonomic group has remained challenging (Börger et al. 2006b). Such analyses have been stymied by a suite of factors that limit the ability of researchers to simultaneously consider multiple influences on space use across large spatial and temporal scales. For example, methodological differences such as differences in the data types (i.e., live-trapping versus radio-tracking) or home range estimators (such as minimum convex polygon (MCP) versus kernel density estimators (KDEs)) used to determine home range area may obscure relationships between phenotypic and environmental predictors and home range area across the range of a species (Börger et al. 2006b; Nilsen et al. 2008; Worton 1989) and across different studies of the same species. Further, the most-often used methods for collecting the location data that are necessary to estimate home range area (live-trapping and radio-telemetry) have historically been both time- and labor-intensive (Kays et al. 2015; Wilmers et al. 2015). Logistical and financial challenges of carrying out such field studies have often limited individual researchers to working at a single site on small numbers of individuals, yielding information about animal space use that can be difficult to synthesize with studies at other locations. The National Ecological Observatory Network (NEON), a long-term project funded by the U.S. National Science Foundation that collects and curates standardized biological data, opens up new opportunities to examine the synergistic influences of multiple phenotypic and environmental factors on animal space use (Dantzer et al. 2023).

Phenotypic attributes of individual animals, including sex, age, body condition, behavior, and neuroendocrine factors, are all expected to influence patterns of animal space use. Sex is likely the most-often tested effect of individual phenotypic variation on home range area in mammals (Clement & Roedder 2021): male mammals often (but not always) have larger home range areas than female conspecifics (Stickel 1968; Wolff 1989; Kalcounis-Rüppell & Ribble 2007; Spencer et al. 1990). This difference in space use between males and females is typically attributed to one of two non-exclusive explanations: body size dimorphism and the promiscuous or polygynous mating systems of most mammalian species (Emlen & Oring 1977; Wolff 1989). Larger animals (typically males in mammalian species with sexual size dimorphism, but see Tombak et al. 2024) often range over larger areas (Spencer et al. 1990); however, sexual size dimorphism has also been associated with non-monogamous mating systems (Andersson 1994), and mating systems can influence space use (Clutton-Brock 1989; Emlen & Oring 1977). In polygynous mating systems, which are common in mammals (Waterman 2007), males are expected to range over larger areas to find and access the home ranges of multiple females, while females are expected to use and defend smaller areas (which would then be considered territories (Emlen & Oring 1977)). The body condition of animals can also affect space use behavior, although it is more often evaluated in relation to dispersal than home range area (reviewed by Clobert et al. 2009). In relation to dispersal, better body condition typically correlates with an increased likelihood to disperse (Holekamp 1986), or increased dispersal distance. Body condition would be expected to influence home range area in a similar way: animals with good body condition should be able to use larger areas than animals in poor condition (Fokidis et al. 2007).

Environmental conditions, such as population density and habitat type, have also been found to affect space use behavior. Typically, population density is negatively correlated with home range area (for example, Šálek et al. 2015; Schradin et al. 2010), and at increased population densities, animals may also exhibit increased home range overlap with neighbors and/or increased territoriality (Wolff 1989). For animals that use multiple types of habitat, home range area may vary with habitat type (e.g., Ofstad et al. 2016). However, it can be difficult to disentangle the effects of habitat type and population density on home range area as these factors often covary (Stickel 1968; Efford et al. 2016). Finally, for widely distributed species, latitude (or factors associated with latitude, such as temperature and resource availability) may affect space use (Gonzalez-Borrajo et al. 2017; Mattisson et al. 2013; Morellet et al. 2013). For example, Gompper & Gittleman (1991) found that the home range area of small carnivores increased with increasing latitude, likely because decreasing resource availability at higher latitudes necessitated the use of larger areas to obtain the resources needed to meet basic needs.

Here we leverage the power of the large temporal and spatial scale of data collected by the National Ecological Observatory Network (NEON) to simultaneously consider the influences of multiple phenotypic and environmental factors on home range area in the most abundant and widely distributed genus of rodents in North America, *Peromyscus* (deer mice). NEON is a nation-wide network of field sites where a variety of ecological and organismal data are being collected over a 30-year period using standardized methodologies. NEON enables comparisons across large temporal and spatial scales; for example, examining the relationship between niche overlap and latitude in rodents (Read et al. 2018). NEON includes 47 terrestrial sites and small mammal live-trapping data are collected at the 44 of these sites (sites in Hawaii and Puerto Rico are excluded). *Peromyscus* is an ideal system for examining the effects of multiple factors on space use: these mice are widely distributed across North America, occur in diverse types of habitat, are relatively short-lived, and typically use small spatial areas (Bedford & Hoekstra 2015) that are often smaller than the NEON sampling grids. Further, while these animals are generally abundant (*Peromyscus* are often the most common mammal in an area), that abundance can vary dramatically across time and space. The dataset that we use here includes captures from the 36 NEON sites with sufficient *Peromyscus* captures for the calculation of a home range, spanning almost 20 degrees of latitude and 10 years, in three macrohabitat types (forest, grassland, and shrubland). The remarkable spatial and temporal replications of the NEON dataset allow us to simultaneously investigate the role of factors both intrinsic (sex and body condition) and external to individual animals (density, habitat type, and latitude), as well as interactions among these factors, on space use in *Peromyscus*. We predicted that males would have larger home range areas than females, that home range area would be negatively correlated with animal density but positively influenced by body condition, that home range area would increase with latitude, and that habitat type would affect home range area.

## Materials and methods

### NEON data

NEON collects small mammal capture data on 1 ha grids set with 100 Sherman traps at 10 × 10 m intervals. Each terrestrial NEON site contains 3–8 small mammal trapping grids and each grid is sampled in 4–6 bouts each year, with bouts taking place over either 1 night (in the case of “diversity grids”) or 3 nights (in the case of “pathogen grids”). When individuals from target species are captured, they are weighed, sexed, measured, and identified to genus, and when possible, to species, before being tagged with a unique identifier (either an ear tag or PIT tag, described in the NEON protocol) and released. Details on the NEON small mammal sampling protocol can be found at: https://data.neonscience.org/data-products/DP1.10072.001.

We retrieved all small mammal capture data available from NEON in January 2023 for use in our analysis (NEON 2024a, NEON 2024b). This initial dataset contained capture data on 169 species from 46 sites from 2013 to 2022. We filtered this initial dataset to include only captures of *Peromyscus* (*n* = 71,945). We conducted our analyses at the genus, rather than species, level for two reasons. First, the original NEON small mammal sampling protocol (Paull et al. 2014) predates a taxonomic revision of *P. maniculatus *sensu lato, which elevated multiple clades within *P. maniculatus *sensu lato to species status (Boria & Blois 2023; Bradley & Lindsey 2019; Greenbaum et al. 2019). Thus, all of these now-recognized species are coded as *P. maniculatus* in the NEON data set. Second, morphological similarities between some distantly-related species lead to difficulty in reliably distinguishing some pairs of syntopic *Peromyscus* species in the field (e.g., *maniculatus*/*leucopus*, *maniculatus*/*keeni*, and *leucopus*/*gossypinus*). For example, *Peromyscus* captured at some NEON sites have been split relatively evenly between *maniculatus* and *leucopus* by field identification, but genetic analysis has failed to document the presence of *maniculatus *sensu lato at these sites (Steger et al. 2024). Because our predictions about space use are the same for all species included in our study, we condensed data to the genus level to avoid any impact that misclassification of species might have on our results. We removed records of *Peromyscus* species that exhibit some degree of social and/or genetic monogamy: *californicus*, *eremicus*, and *polionotus* (Dewsbury 1981; Kalcounis-Rüppell & Ribble 2007). Not only is monogamy an atypical mating system for the genus, monogamy would also be expected to influence predictions about sex differences in home range area (Bester-Meredith et al. 2017; Emlen & Oring 1977; Kalcounis-Rüppell & Ribble 2007). These three species have relatively limited distributions and are morphologically easy to distinguish from other *Peromyscus* species (*californicus* and *polionotus* are the largest and the smallest members of the genus, respectively). Thus, unlike some other species of *Peromyscus*, these three species can be reliably identified and removed from the dataset. After the removal of *californicus*, *eremicus*, and *polionotus*, which constituted < 1% of *Peromyscus* captures (696 of 71,945), we were left with the following field-identified *Peromyscus* species in our dataset (ordered from the largest to the smallest sample size): *maniculatus *sensu lato (33,227 captures, 46%), *leucopus* (25,235 captures, 35%), *gossypinus* (4,511 captures, 6%), *boylii* (2,559 captures, 3.5%), *keeni* (1,450 captures, 2%), *truei* (1,364 captures, 2%), and *attwateri* (48 captures, < 1%). Although we conducted our analysis at the genus level, we present data on field identification of species here for transparency, and note that more than 80% of *Peromyscus* captures included in our study were field-identified as either *maniculatus *sensu lato or *leucopus*.

### Assignment of sex

Although there are clear differences in external genitalia between male and female *Peromyscus*, misidentifications can occur, especially for non-reproductive animals. In our analysis, we assigned the sex of each individual as it was noted in the NEON database for the majority of its capture events. For example, if an animal was noted as a male for more captures than it was noted to be a female, we considered it to be a male, and vice versa. We also used ‘pregnancy status’ in the NEON database to further identify females. We considered any individual that was ever noted to be pregnant as a female, regardless of the number of times that it was recorded as a male. This sex assignment procedure was applied to all animals in our analysis, resulting in a dataset composed of 1,148 females and 1,272 males.

### Determining animal life stage

We included only captures of adult animals in our analysis because space use by juveniles may reflect use of the mother’s home range, dispersal, or other developmental processes that do not reflect typical space use. Thus, if an animal was captured when it was both a juvenile and later as an adult, we considered in our analysis only its captures as an adult.

Each capture was assigned a life stage by NEON field technicians, but in our analysis, we assigned a life stage to each capture based on body mass to avoid relying on subjective criteria, such as stage of the post-juvenal molt, which may be applied unevenly across observers. To assign life stage based on body mass, we first distinguished between species that are typically larger and those that are typically smaller. If we had used body mass cutoffs to distinguish life stages at the genus level, we might have assigned captures of small species as juveniles of large species. We applied one size cut-off to field-identified species that are ‘small’ and a different cut-off to species that are ‘large’, and determined which species are ‘small’ and which are ‘large’ based on previously published data on body mass of adult mice (Derrickson 1988; Drickamer & Bernstein 1972; Layne 1968; McCabe & Blanchard 1950; Pournelle 1952; Wolff et al. 1988). ‘Small’ species included field-identified *maniculatus *sensu lato and *leucopus*, and for these purported species, we assigned a ‘subadult’ life stage to captures of animals < 16 g and an ‘adult’ life stage to captures of animals 16 + g. ‘Large’ species included field-identified *keeni*, *truei*, *gossypinus*, *attwateri*, and *boylii*, and here we assigned a ‘subadult’ life stage to captures < 19 g and an ‘adult’ life stage to captures 19 + g. Mass records differed significantly between the ‘small’ and ‘large’ species categories we used (T test: *t* = 40.749, *p* value < 0.0001; Figure S3A), justifying our assignment of each field-identified species as a ‘small’ or ‘large’ species. Our data consisted of more than 80% individuals from ‘small’ species.

In some cases, individuals were assigned to the ‘adult’ life stage based on body mass at an early capture, but in later captures, the mass was not recorded (‘NA’ entered for mass in at least one capture for *N* = 1,146 individuals), or individuals lost mass and dropped below the adult mass cut-off in later captures (*N* = 63 individuals). To account for the missing data and weight loss, we assigned an ‘adult’ life stage to all captures that occurred after the first time an individual's mass was above the ‘adult’ threshold.

### Computing body condition

To quantify body condition of each individual, we calculated the mean body mass (g) and mean hindfoot length (mm) for each individual from all of its captures (Schulte-Hostedde et al. 2005). To avoid the confounding effects of pregnancy on body condition estimates, we excluded from this calculation any captures when an individual was pregnant or when pregnancy status was listed as ‘unknown’. We then regressed the mean mass against the mean hindfoot length using the lm() function in R ‘base’, and assigned the residuals of this regression to each individual in our dataset as their body condition, resulting in a single condition estimate for each animal. We conducted these regressions and body condition assignments separately for the ‘small’ and ‘large’ species (see explanation of these in the ‘Determining animal life stage’ section above) to account for differences in mass and hindfoot length between large and small species (Figure S3B,C).

### Habitat types

NEON assigns each plot a vegetation type based on National Land Cover Database (NLCD) classes, using remote-sensed Landsat data. We used these NLCD classes to assign the vegetation type to each animal’s home range (all home ranges within a plot had the same vegetation type). To examine the effect of vegetation type on home range area, we grouped NLCD classes into three categories to ease analysis and biological interpretation. The three categories were: forest (NLCD classes 'deciduousForest', 'mixedForest', 'evergreenForest', and 'woodyWetlands'), grassland (NLCD classes 'grasslandHerbaceous', 'cultivatedCrops', and ‘pastureHay’) and shrubland (NLCD class 'shrubScrub').

### Calculating animal density

To estimate the density of congeners that each focal individual experienced, we calculated the minimum number of *Peromyscus* individuals known alive (MNKA) at each plot on each sampling event (Paull 2022). For this calculation, individuals were considered to be “known alive” for all sampling events between their first and last captures at the sampling plot. We assigned each individual in our dataset a ‘meanMNKA’, which was the mean of all MNKA values for the plot in which the focal individual was captured, during all sampling events between the first and last capture of the focal individual. MNKA provides a generally reliable index of population size that is suitable for our purposes as we are interested in how density may influence home range area, rather than the actual density itself. While MNKA is known to underestimate true population size, it is also strongly and positively correlated with population estimates derived from statistical models that incorporate probability of capture (Slade and Blair 2000). Further, estimating density with these types of models typically requires species-specific capture probabilities (see Parsons et al. 2023 for a NEON-specific example), and species identifications in our dataset are not reliable, as described above. Thus, our estimate of MNKA combines all *Peromyscus* species captured at a plot.

### Removing outliers

Some of the capture records had weights above 50 g (*N* = 8, out of 23,959 captures of individuals captured at least 5 times from focal *Peromyscus* species) or hindfoot length greater than 28 mm (*N* = 7, out of 23,959 captures). These values are unlikely for the species in our dataset, therefore we replaced the values for weight and hindfoot length with NA for those 15 records and included them only for the home range analysis but not the body condition analysis. We also removed one individual that was recorded as 9 g, which is an unrealistically low weight for an adult. In addition, we removed one individual with inconsistent pregnancy status (i.e., consecutive days fluctuating between positive and negative pregnancy status).

### Home range area calculation

To calculate home range area, we computed the utilization distribution of each animal and considered the 50% kernel area as the home range area. We only included in this analysis animals that had five or more capture events from at least three unique locations, which reduced the number of captures in our analysis from 23,959 to 18,148. We used the functions ‘kernelUD()’ and ‘kernel.area()’ from the R package ‘adehabitatHR’ (Calenge 2006) to calculate 50% kernel density estimate home range areas.

We focused our analysis of home range area on animals that had five or more captures as adults to ensure a reliable home range calculation and based on a rarefaction analysis that determined that five locations, as a minimum number of captures, provided sufficient information to obtain an accurate home range area using a utilization distribution (or kernel density estimation) approach (see Supporting Information). The rarefaction analysis further showed that using a minimum convex polygon (MCP) to calculate home range area was not a reliable method for animals with too few captures (as found by others: Börger et al. 2006a, b; Börger et al. 2008; Socias‐Martínez et al. 2023) and so we only used a utilization distribution (KDE) approach here to calculate home range area. Areas of some home ranges might be underestimated for animals living near the edge of a trapping grid; however, we have no reason to expect that such underestimates are distributed in a non-random way across factors of interest (e.g., that males or females are more/less likely to be at the edge of a sampling plot).

After removing individuals that were captured fewer than five times as adults, we calculated the home range area of 2,420 individual *Peromyscus* based on 18,148 capture events: 1,016 *leucopus*, 881 *maniculatus *sensu lato, 251 *gossypinus*, 111 *boylii*, 59 *truei*, 26 *keeni*, and 2 *attwateri*, as well as 50 identified as either *leucopus* or *maniculatus*, 11 identified as either *gossypinus* or *leucopus*, and 13 identified as *Peromyscus* without a species designation. These species identifications are based on field records rather than genetic information and may be somewhat unreliable due to known concerns that are described above. We condensed the data to the level of genus due to those concerns, but note that ~ 80% of individual mice included in the analysis were field-identified as either *maniculatus *sensu lato or *leucopus.*

### Statistical analysis

To determine what factors impact home range area in *Peromyscus*, we used a statistical model selection approach in which we compared Generalized Mixed Models (GLMMs) with different interaction terms between the factors of interest (see Supporting Information for a list of the models tested and for their comparisons). We only included interaction terms that had biological meaning (Johnson & Omland 2004). In all models, home range area (*N* = 2,420) was the response variable. Explanatory variables included sex, body condition, vegetation type, animal density (meanMNKA), and latitude as fixed effects. All models also included year and site as random effects to account for variation across years and sites in the model. All models were fitted with a Gamma distribution and log link function using the ‘lme4’ R package (Bates et al. 2015) and analysis of deviance tables were obtained using the Anova() function in the ‘car’ R package (Fox & Weisberg 2019). We examined if models met all statistical assumptions (linearity, homogeneity of variance, etc.) using the check_model() function in the package ‘performance’. Finally, we compared the AIC values of all models examined using the compare_performance() function in the package ‘performance’ (Lüdecke et al. 2021). We selected the best fit model based on AIC weight.

## Results

**Fig. 1:**
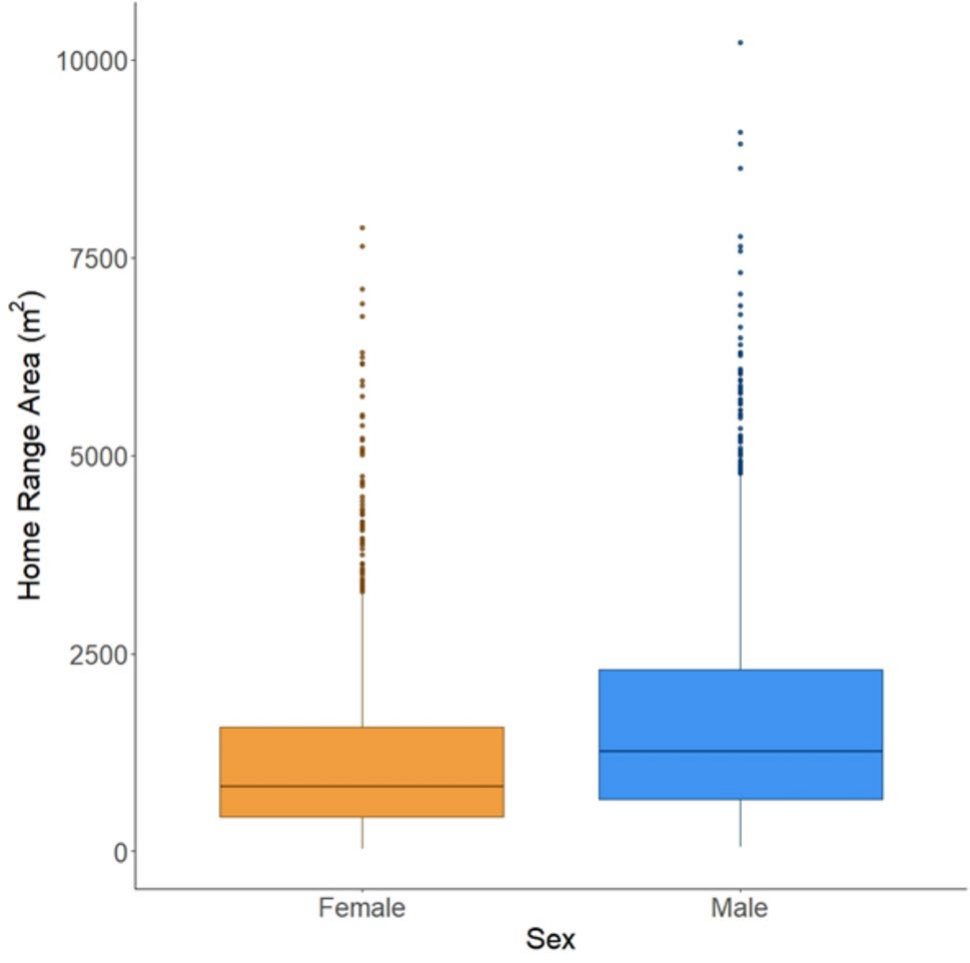
Home range area (m^2^) by sex—males in blue and females in orange. Horizontal lines indicate the median, boxplots indicate the interquartile range, vertical lines extend to 1.5 times the interquartile range, and points indicate outliers

**Fig. 2:**
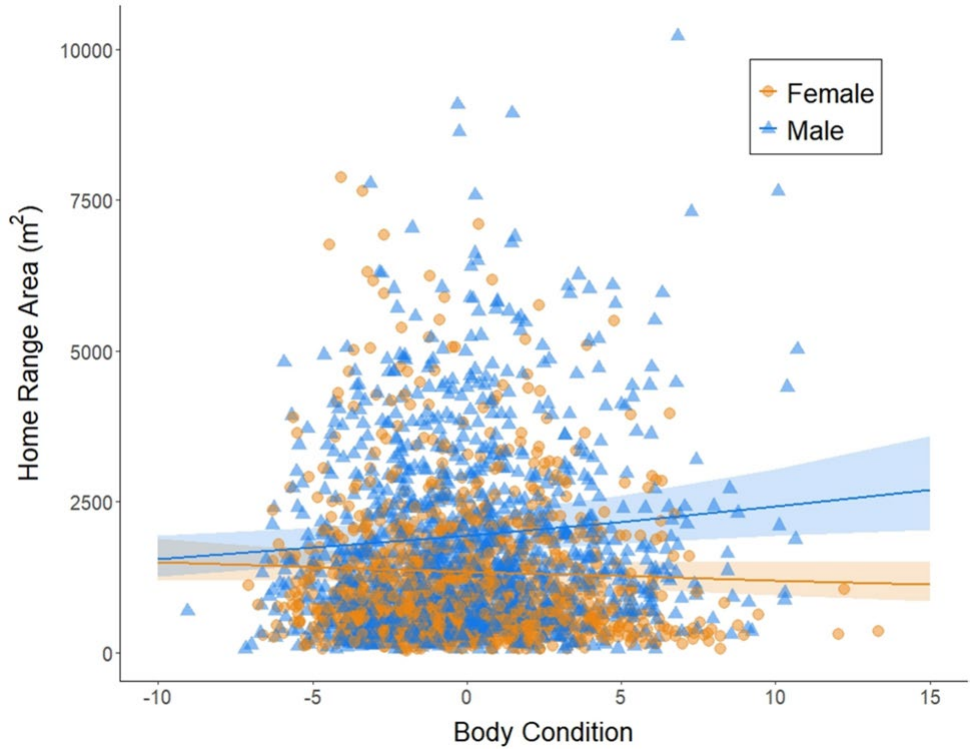
Home range area (m^2^) as a function of body condition for males (blue triangles) and females (orange circles). Each point is an individual mouse and the lines are the predicted values from the statistical model, with confidence intervals as shaded areas around the lines

Our dataset consisted of home range areas calculated for 2,420 adult *Peromyscus* (1,148 females and 1,272 males), based on 18,148 capture events between 2013 and 2022 at 36 sites between 28 and 47.2 degrees latitude. The mean home range area we calculated was 1,463 m^2^ (SD = 1,348 m^2^, range = 29.5–10,219.8 m^2^). Animal density (‘meanMNKA’) was on average, across all individuals, 21.98 *Peromyscus* known alive/1 hectare trapping grid (SD = 13.4, range = 1.4–77.3). Of the 12 models we tested (specified in Supporting Information), the best fit model included interaction terms between sex and body condition, habitat type and latitude, latitude and animal density, habitat type and animal density, and the three-way interaction of habitat type, animal density, and latitude (AIC weight = 0.98, Table 1, for all AIC values, see Table S1). The main factors that had a significant impact on home range area were sex, habitat type, latitude, and animal density (Tables 1, S2). Interestingly, body condition by itself did not have a significant impact on home range area, it only influenced home range area when considering its interaction with sex. Overall, males had larger home ranges than females (Fig. 1, Tables 1, S2) and as male body condition improved, home range area increased (Fig. 2). In contrast, as female body condition improved, home range area decreased (Fig. 2).

**Table 1:** Analysis of deviance of the best fit model. Significant effects (*p*<0.05) denoted in bold

Effect	Chisq	Df	*P*-value
Sex	**138.188**	**1**	** < 0.0001**
Body Condition	1.059	1	0.303
Habitat type	**6.657**	**2**	**0.036**
Latitude	**9.257**	**1**	**0.002**
Animal density (meanMNKA)	**286.201**	**1**	** < 0.0001**
Sex x Body Condition	**10.287**	**1**	**0.001**
Habitat type x Latitude	5.840	2	0.054
Latitude x Animal density (meanMNKA)	**14.731**	**1**	**0.0001**
Habitat type x Animal density (meanMNKA)	5.859	2	0.053
Latitude x Habitat type x Animal density (meanMNKA)	**19.242**	**2**	** < 0.0001**

**Fig. 3:**
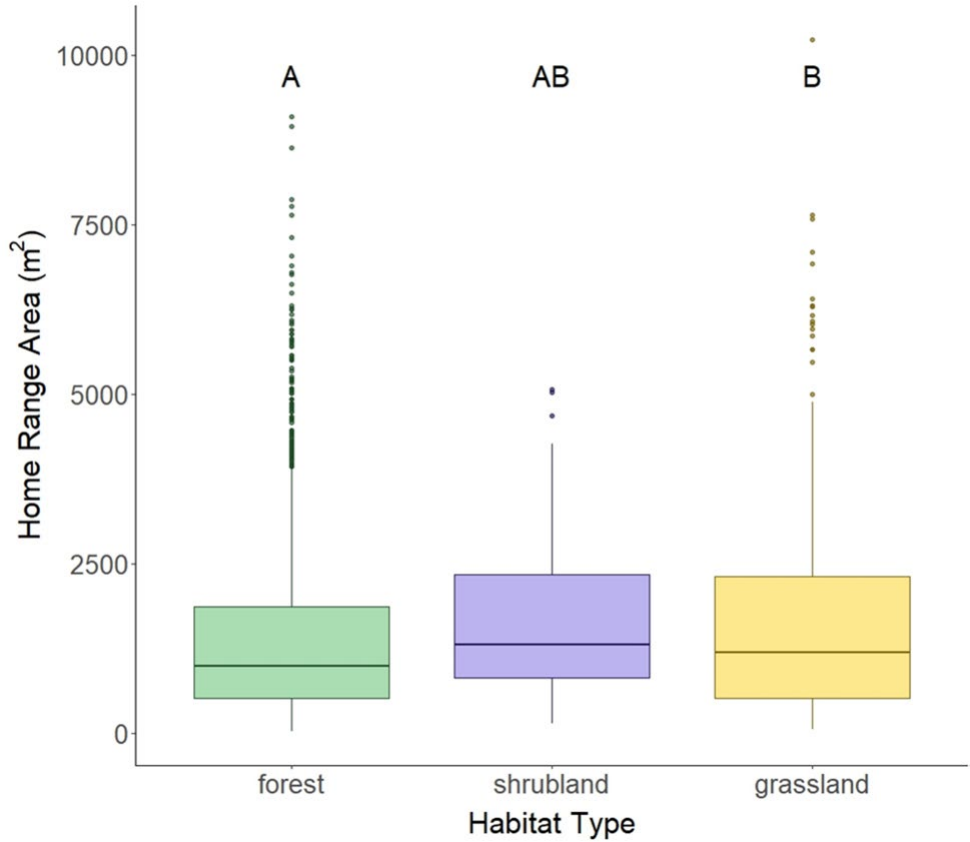
Home range area (m^2^) by habitat type—forests in green, shrublands in purple, and grasslands in yellow. Horizontal lines indicate the median, boxplots indicate the interquartile range, vertical lines extend to 1.5 times the interquartile range, and points indicate outliers. Boxes that do not share a letter above them are statistically significantly different according to a post hoc Tukey test

Home range area varied by habitat type, with the smallest areas used by mice in forested habitat and the largest areas used in grasslands. Home range areas in shrublands were intermediate and not significantly different from home range areas in either forests or grasslands (post hoc Tukey test, Fig. 3, Tables 1, S2).

**Fig. 4:**
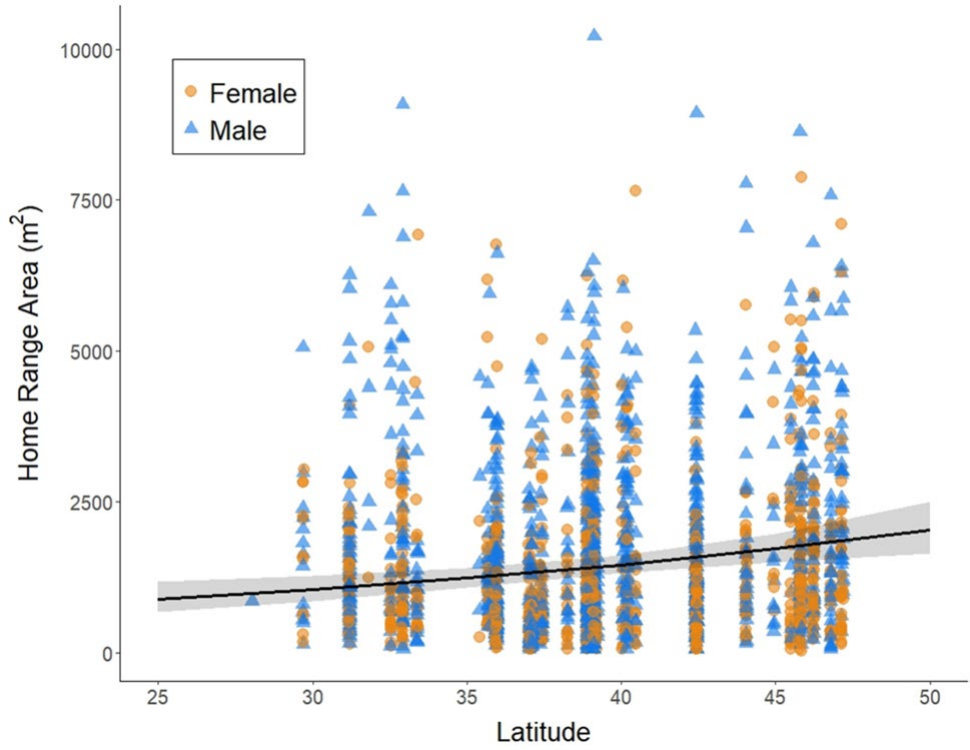
Home range area (m^2^) as a function of latitude for males (blue triangles) and females (orange circles). Each point is an individual mouse and the line is the predicted values from the statistical model, with confidence intervals as shaded areas around the line

**Fig. 5:**
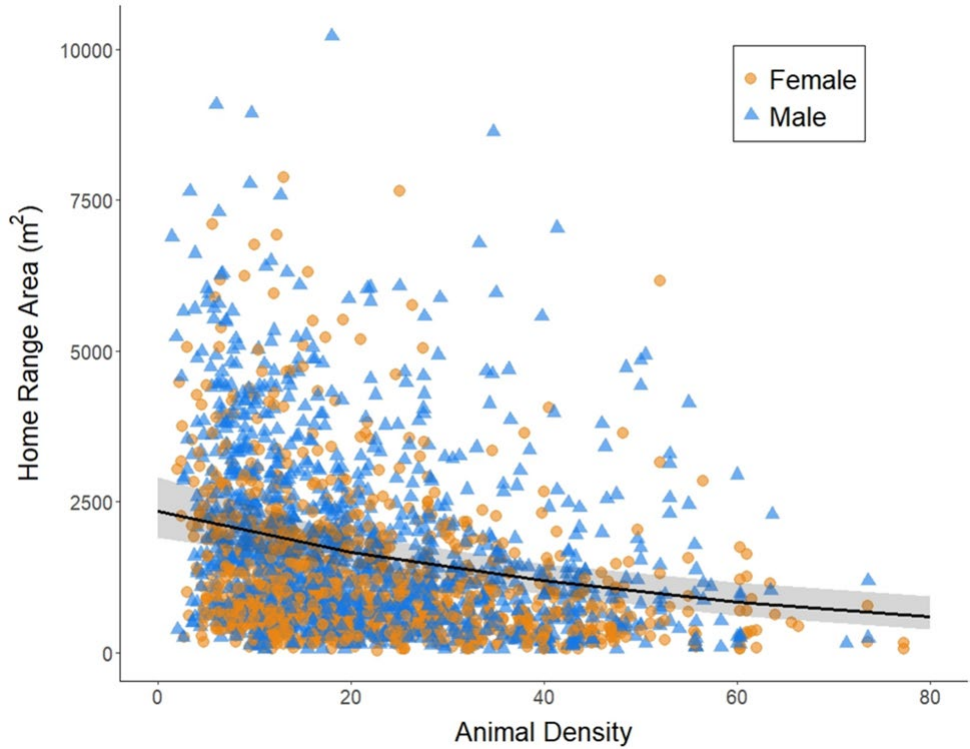
Home range area (m^2^) as a function of animal density for males (blue triangles) and females (orange circles). Each point is an individual mouse and the line is the predicted values from the statistical model, with confidence intervals as shaded areas around the line

Home range area increased with latitude (Fig. 4, Tables 1, S2) and decreased with animal density (Fig. 5, Tables 1, S2). There was a significant interaction between latitude and animal density (Tables 1, S2), which means that the effect of animal density on home range area differs across latitudes. Latitude and animal density are positively correlated with one another (correlation coefficient = 0.28, see Figure S4), which might suggest that latitude explains animal density rather than home range area. However, the fact that the relationship between home range area and latitude is in the opposite direction of the relationship between home range area and animal density indicates that both factors have an important impact on home range area, regardless of the impact of latitude on animal density.

**Fig. 6:**
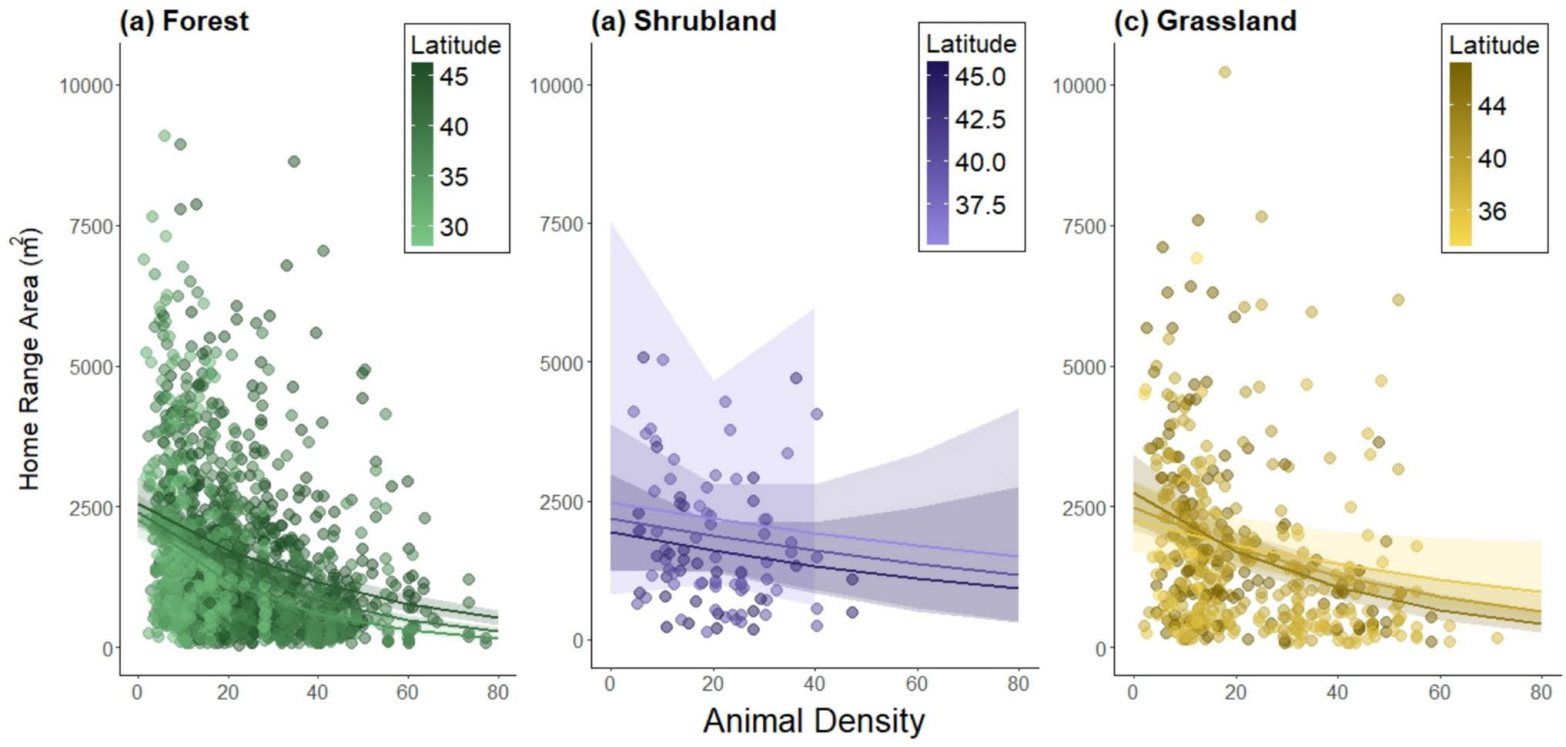
Home range area (m^2^) as a function of animal density in (**a**) forests (green), (**b**) shrublands (purple), and (**c**) grasslands (yellow). Each point is an individual mouse and darker points are from higher latitudes (see color scale in each panel). The lines show relationships for three latitude ranges, determined by the emmeans() function. Note that the statistical model treats latitude as a continuous variable but three discrete lines are shown to assist the interpretation of the statistical interaction between animal density, latitude, and habitat type

Finally, we found a significant interaction between latitude, animal density, and habitat type (Tables 1, S2), which means that the relationship between home range area, latitude, and animal density differs across habitat types. Indeed, in forests, home range areas are larger at higher latitudes, and the decline in home range area with increasing animal density is slightly more steep at lower than higher latitudes (Fig. 6a). In contrast, home range areas in shrublands are larger at lower latitudes than at higher latitudes. The decline of home range area with animal density in shrublands does not seem to differ across latitudes, but the rate of this decrease (slope of the line) is smaller than in forests (Fig. 6b). Finally, in grasslands, home range areas are larger in high compared to low latitudes when animal density is low, but as animal density increases, home range areas decrease as latitude increases. Thus, the rate at which home range areas decrease with animal density (slope of the line) is greater in high latitudes than in low latitudes in grassland habitats (Fig. 6b).

## Discussion

Despite long-standing interest in the simultaneous effects of factors intrinsic to individuals (phenotypic traits) and the socioecological environments in which they exist on space use by animals, integrative studies have been hampered by both small sample sizes and methodological differences across studies. Here, we leveraged the power of replication across time and space by the National Ecological Observatory Network (NEON) to investigate multiple simultaneous influences on space use by *Peromyscus* mice, analyzing the home range areas of almost 2,500 animals trapped across 10 years and almost 20 degrees of latitude. Because NEON employs consistent sampling methodologies across sites, we can be confident that observed differences across latitudes and habitat types are not mere artifacts of variation in sampling methods across studies and researchers. Importantly, the large sample size gives us the necessary statistical power to conduct robust statistical tests that include interaction terms (see supporting information for full list of models tested). The top-ranked model in our analysis included statistically significant interactions between both phenotypic traits (sex x body condition) and factors external to the animal (latitude x habitat type x animal density) that had an effect on space use (Table 1).

Considering the effect of individual phenotype on home range area, our finding that male *Peromyscus* have larger home range areas than females (Fig. 1) is consistent with previous studies (as reviewed by Kalcounis-Rüppell & Ribble 2007). However, the significant interaction we found between sex and body condition reveals additional nuance: as body condition increases, male mice use larger home ranges, whereas female mice use smaller home ranges (Fig. 2). This result is consistent with established theory about space use by male and female mammals: Emlen and Oring (1977) posited that the distribution of female mammals across a landscape should be influenced by the distribution of resources, and the distribution of males should be influenced by the locations of females. Under Emlen and Oring’s theory, the male strategy for maximizing reproductive success is to overlap the home ranges of more females and sire offspring by as many females as possible, while the female strategy is to obtain the resources needed to produce their offspring as efficiently as possible. This classic theory of differential space use by the sexes is almost certainly too simplistic (for example, multiple mating by both sexes is now known to be common across mammals), but our results are consistent with expectations arising from it: as body condition increases, male *Peromyscus* range more widely (Fig. 2). Meanwhile, our results suggest that females with higher body condition do not need as much space to meet their energetic needs, due either to their internal energetic reserves, or because they are stronger competitors for better quality habitat where they can obtain sufficient energy over a smaller area. Importantly, we removed from our analysis any females who were pregnant, so the higher values of female body condition do not reflect temporary mass gains (and inflated body condition scores) due to pregnancy. Future work that relies on more spatially specific habitat information, as well as reproductive success, which both are not available as part of NEON data, would allow a more detailed investigation of this hypothesis.

Individual animals exist within a spatially and temporally variable socioecological environment. We found that factors external to the individual animal, including habitat type, animal density, and latitude, all had important effects on home range area. Home range areas were the smallest in structurally more complex forested habitat types and the largest in less complex grassland habitat types, with intermediate home range areas in shrubland habitat (Fig. 3), a result consistent with previous findings in other mammalian groups (e.g., Ofstad et al. 2016 for ungulates). Habitat complexity can impact foraging strategies, influencing how animals use space (Marines-Macías et al. 2018; Rader & Krockenberger 2006). Furthermore, use of arboreal habitat may influence estimates of home range size because the vertical aspect of space use is not accounted for in traditional 2D home range estimates (Heit et al. 2021). We further replicated the widely-accepted relationship between the density of competitors (including both conspecifics and congeners) and home range area (e.g., Efford et al. 2016): as animal density increases, home range area decreases (Fig. 5). Similarly, we replicated previous findings about the effect of latitude on home range areas (e.g., Gompper & Gittleman 1991 for mesocarnivores): *Peromyscus* home range areas increased with increasing latitude, likely because home ranges tend to be larger when resources are sparse. As ecosystem productivity (net primary productivity, NPP) drops with increasing latitude (for example, Kicklighter et al. 1999; Gillman et al. 2015), the food resources available to animals such as *Peromyscus* would also be expected to decline. This link between latitude and home range size is consistent with the relationship between home range size and seasonality of resources across mammalian species (Broekman et al. 2024).

While our results confirmed several known relationships between home range area and socioecological factors, the novelty and the strength of our work lie in the large sample size that allowed us to examine more nuanced interactions among these factors. Of particular interest is the complex and unanticipated interaction between latitude, animal density, and habitat type (Fig. 6). When considering the main effects of these socioecological variables in isolation, home range area increased with increasing latitude (Fig. 4), decreased with increasing conspecific density (Fig. 5), and varied among habitat types (Fig. 3). Thus, one might expect that home range area would be the largest at higher latitudes when animal density was low (due to few resources and little competition for space), and the smallest at lower latitudes when animal density was high (due to high resource availability and increased competition for space). This is exactly that pattern that we observed in forested habitat (Fig. 6a). However, in shrublands, we observed that home ranges were larger at lower latitudes (Fig. 6b), and in grasslands, the slope of the relationship between density and home range area varied across latitudes (Fig. 6c). There are several possible explanations for these varying patterns. One possibility is that resource availability for *Peromyscus* in non-forested habitats may respond in different ways to latitudinal variation. For example, the factors that influence allocation of NPP to above- and below-ground components are complex, but in general, relative allocation to below-ground NPP increases with latitude and varies across biomes and with varying precipitation (Gherardi & Sala 2020). Such patterns may affect what resources are available to animals that primarily forage above ground. Another possibility is that predation pressure, which varies across habitat types and is predicted to affect space use (Ofstad et al. 2016), may also vary with latitude in unanticipated ways. Some of these questions are amenable to further investigation using other NEON data sets.

Our work shows that data from large ecological networks can be used to reveal important behavioral questions that have long eluded investigators. With a large sample size that spans the continent spatially and an entire decade temporally, we were able to uncover novel relationships between animal space use and both phenotypic and environmental factors. Thus, large ecological monitoring networks can be used not only to uncover changes in ecological patterns, but also to examine how organismal biology will change as our world continues to be impacted by human activities.

## Supplementary Information

Below is the link to the electronic supplementary material.

Supplementary file 1 (DOCX 2727 KB)

## Data Availability

All data (downloaded from the NEON repository) are available on Github: https://github.com/seanofallon/neon-mice_home-ranges.

## Code availability

Analysis code is available on Github: https://github.com/seanofallon/neon-mice_home-ranges.
