# Supplementary material for: Uncovering multiple influences on space use by deer mice using large ecological networks
Source: Oecologia. 2025 Jun 4;207(7):98. doi: 10.1007/s00442-025-05731-2 (PMC12133976; doi:10.1007/s00442-025-05731-2)
Supplement: Supplementary file 1 — (DOCX 2727 KB) [file 442_2025_5731_MOESM1_ESM.docx]

**Supplementary Materials for:**

**Uncovering multiple influences on space use by deer mice using NEON data**

**Table of contents:**

1. Rarefaction of home range - effect of sample size on HR size
2. Body sizes of small and large *Peromyscus* species
3. Model selection for *Peromyscus*
4. Map of NEON field sites used in analysis

_______________________

**1. Rarefaction of home range**

**Number of captures used to estimate home range:**

To determine the smallest number of captures that could be used to obtain a reliable estimate of home range size, we ran a sub-sampling analysis. In this analysis we only used individuals that had 10 or more captures (N=93) and the largest number of captures was 25. We calculated an ‘observed’ home range size using all available capture information for each individual with either a minimum convex polygon (MCP) or a utilization distribution (UD) using the functions mcp.area() and kernel.area() in the R package ‘adehabitatHR’ {ref}. We then sub-sampled (without replacement) the captures of each individual to take 5,6,7,8,or 9 random captures from those available for each individual. We sampled each individual and each sample size 50 times and calculated home range sizes using the sub-sampled data with both MCP and UD home range estimates. We scaled each home range obtained from the sub-sampled data to the ‘observed’ home range (which was calculated using all available data points for each individual) by dividing the sub-sampled home range by the observed home range. A scaled home range value equal to 1 means that the home range from the sub-sampled data is identical to the home range calculated from all samples available for the individual. Values smaller than 1 indicate that the sub-sampled data underestimates home range size and values larger than 1 indicate that the sub-sampled data overestimates the observed home range size. To examine the impact of number of data points (captures) on our ability to estimate an individual's home range, we averaged the scaled home range (MCP or UD) for each individual over the 50 runs of the simulation for each number of subsampled captures (i.e., 5,6,7,8,and 9). We thus obtained 93 values of scaled home range sizes for each sample size we examined (5-9) for both MCP and UD home range estimates (Figure S1).

**Figure S1:** Scaled home range sizes from the sub-sampling analysis using MCP (left) or UD (right) estimates for home range. We compared 5 different sample sizes (5-9) depicted on the x axis. Each individual was sub-sampled 50 times for each sample size and the scaled home range sizes for each individual was averaged over the 50 simulation runs. Boxplots show the averaged scaled home ranges for each sample size for the 93 individuals we used in this analysis.


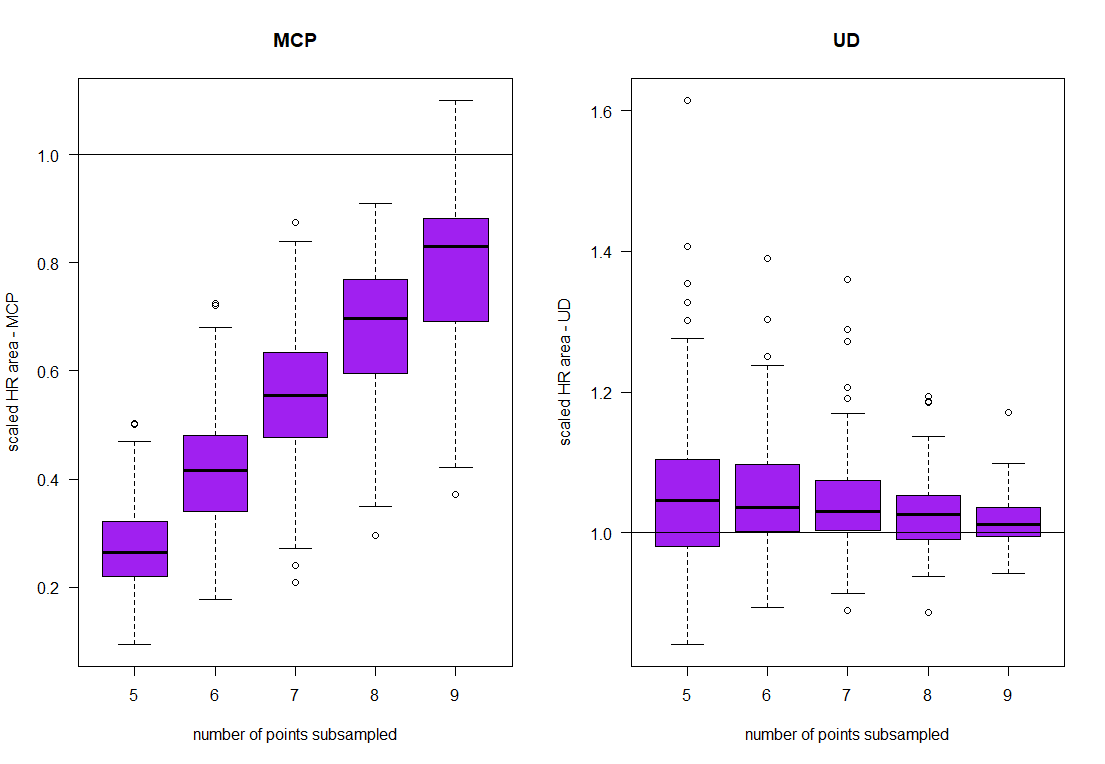


**Figure S2:** A comparison of MCP and UD home range estimates for individuals with 10 or more captures.

**
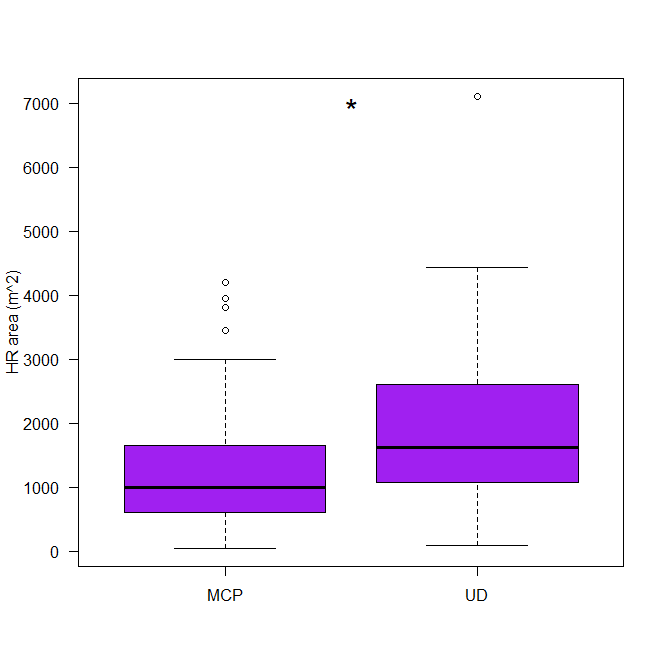
**

*Results:* We found that when using an MCP estimate, as the number of data points (captures) increased we obtained home range size estimates that were closer to those observed when using all available data points. In contrast, the UD estimator for home range size was consistently similar to the one we observed when using all data points available for each individual, regardless of how many data points we used. Even when using only 5 data points, the home range sizes that we estimated were very similar to those we found when using 10 or more observations. The main impact that decreasing the sample size had on the home range size calculated with a UD estimate was a decrease in accuracy - the spread of estimates around the observed value was larger for smaller samples.

*Conclusions:* Consistent with past investigations that compared different estimation approaches of home range size (Socias-Martinez et al., 2023, cited in main text), we found that the MCP measure of home range size is highly sensitive to the number of repeated captures for each individual. However, estimating home range size using UD was not very sensitive to sample size and even sub-samples as small as 5 provided a reliable estimate of home range size.

Given this investigation we decided to only use the UD estimation of home range size in our study - to avoid biases in home range size that might result from differences in sample size across individuals. Furthermore, because a sub-sample of 5 data points provided a reliable estimate of home range size, that was similar to a home range size calculated with 10 or more points, we included in our analysis all animals that had 5 or more re-captures.

**2. Body sizes of small and large *Peromyscus* species**

**Figure S3:** Small (*maniculatus*, *leucopus*) and large (*keeni*, *truei*, *gossypinus*, *attwateri*, and *boylii*) *Peromyscus* species had different mass (left). Body condition was calculated separately for big and small species because the relationship between mass and hindfoot length differed for big (middle) and small (right) species.

**
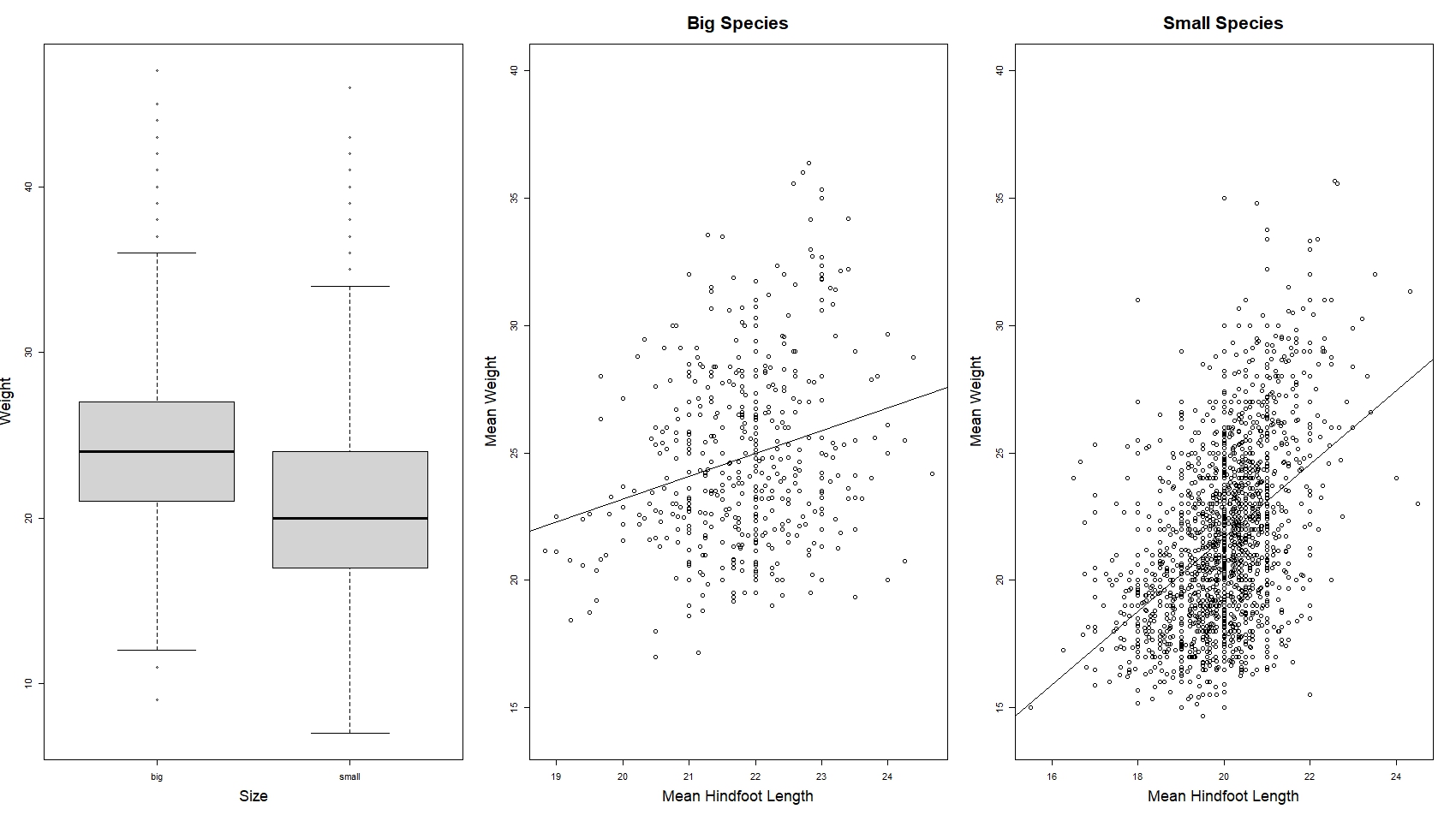
**

**3. Model comparison for *Peromyscus*:**

# no interaction terms:

m1=glmer(UD ~ sex + vegType + meanMNKA + latitude + bodyCondition +

(1 | year) + (1 | site), data = ao_ranges, family = Gamma(link = "log"))

# models with interactions:

m2=glmer(UD ~ sex + vegType * latitude + meanMNKA + bodyCondition +

(1 | year) + (1 | site), data = ao_ranges, family = Gamma(link = "log"))

m3=glmer(UD ~ sex * bodyCondition + vegType + latitude + meanMNKA +

(1 | year) + (1 | site), data = ao_ranges, family = Gamma(link = "log"))

m4=glmer(UD ~ sex * bodyCondition + vegType * latitude + meanMNKA +

(1 | year) + (1 | site), data = ao_ranges, family = Gamma(link = "log"))

m5=glmer(UD ~ sex + bodyCondition * latitude + vegType + meanMNKA +

(1 | year) + (1 | site), data = ao_ranges, family = Gamma(link = "log"))

m6=glmer(UD ~ bodyCondition + latitude + sex * vegType * meanMNKA +

(1 | year) + (1 | site), data = ao_ranges, family = Gamma(link = "log"))

m7=glmer(UD ~ bodyCondition + sex + latitude * vegType * meanMNKA +

(1 | year) + (1 | site), data = ao_ranges, family = Gamma(link = "log"))

m8=glmer(UD ~ bodyCondition * sex + latitude * vegType * meanMNKA +

(1 | year) + (1 | site), data = ao_ranges, family = Gamma(link = "log"))

m9=glmer(UD ~ bodyCondition * sex * meanMNKA + latitude * vegType +

(1 | year) + (1 | site), data = ao_ranges, family = Gamma(link = "log"))

m10=glmer(UD ~ vegType + sex + latitude * bodyCondition * meanMNKA +

(1 | year) + (1 | site), data = ao_ranges, family = Gamma(link = "log"))

m11=glmer(UD ~ sex * meanMNKA + latitude + bodyCondition + vegType +

(1 | year) + (1 | site), data = ao_ranges, family = Gamma(link = "log"))

m12=glmer(UD ~ sex * meanMNKA * bodyCondition + latitude + vegType +

(1 | year) + (1 | site), data = ao_ranges, family = Gamma(link = "log"))

**Table S1:** Model comparison results for *Peromyscus* genus.

Name | Model | AIC (weights) | AICc (weights) | BIC (weights) | R2 (cond.) | R2 (marg.) | ICC | RMSE | Sigma

--------------------------------------------------------------------------------------------------------------------------

**m8 | glmerMod | 39424.2 (0.982) | 39424.5 (0.982) | 39528.4 (<.001) | 0.212 | 0.168 | 0.053 | 1191.150 | 0.822**

m6 | glmerMod | 39446.2 (<.001) | 39446.4 (<.001) | 39544.6 (<.001) | 0.217 | 0.162 | 0.066 | 1198.529 | 0.823

m3 | glmerMod | 39441.4 (<.001) | 39441.5 (<.001) | 39505.1 (0.851) | 0.209 | 0.157 | 0.062 | 1197.356 | 0.828

m7 | glmerMod | 39432.5 (0.015) | 39432.8 (0.016) | 39531.0 (<.001) | 0.210 | 0.167 | 0.052 | 1196.054 | 0.822

m12 | glmerMod | 39439.9 (<.001) | 39440.0 (<.001) | 39520.9 (<.001) | 0.211 | 0.160 | 0.062 | 1196.564 | 0.824

m9 | glmerMod | 39437.7 (0.001) | 39437.9 (0.001) | 39530.4 (<.001) | 0.206 | 0.157 | 0.058 | 1196.205 | 0.822

m4 | glmerMod | 39438.9 (<.001) | 39439.1 (<.001) | 39514.2 (0.009) | 0.204 | 0.154 | 0.059 | 1197.234 | 0.825

m1 | glmerMod | 39450.9 (<.001) | 39451.0 (<.001) | 39508.8 (0.132) | 0.206 | 0.154 | 0.061 | 1202.246 | 0.829

m11 | glmerMod | 39452.8 (<.001) | 39452.9 (<.001) | 39516.5 (0.003) | 0.206 | 0.154 | 0.062 | 1202.353 | 0.829

m5 | glmerMod | 39452.9 (<.001) | 39453.0 (<.001) | 39516.6 (0.003) | 0.206 | 0.154 | 0.061 | 1202.232 | 0.829

m2 | glmerMod | 39448.8 (<.001) | 39448.9 (<.001) | 39518.3 (0.001) | 0.201 | 0.151 | 0.059 | 1202.463 | 0.827

m10 | glmerMod | 39449.5 (<.001) | 39449.7 (<.001) | 39530.6 (<.001) | 0.201 | 0.158 | 0.051 | 1200.175 | 0.831

**Table S2:** Results of best-supported model (m8 from above table).

| Term (level) | Estimate | Std. Error | t value | Pr(>\|z\|) |
| --- | --- | --- | --- | --- |
| (Intercept) | 7.059 | 0.441 | 16.004 | >0.0001 |
| Body Condition | -0.011 | 0.008 | -1.448 | 0.1477 |
| Sex (male) | 0.367 | 0.031 | 11.738 | >0.0001 |
| Latitude | 0.014 | 0.011 | 1.218 | 0.2232 |
| Habitat Type (shrubland) | 1.563 | 3.103 | 0.504 | 0.6145 |
| Habitat Type (grassland) | -0.349 | 0.906 | -0.386 | 0.6997 |
| Animal Density | -0.087 | 0.012 | -7.136 | >0.0001 |
| bodyCondition : Sex (male) | 0.034 | 0.010 | 3.207 | 0.0013 |
| Latitude : Habitat Type (shrubland) | -0.042 | 0.073 | -0.576 | 0.5647 |
| Latitude : Habitat Type (grassland) | 0.010 | 0.023 | 0.425 | 0.6707 |
| Latitude : Animal Density | 0.002 | >0.001 | 5.118 | >0.0001 |
| vegType (shrubland) : Animal Density | 0.093 | 0.121 | 0.763 | 0.4455 |
| vegType (grassland) : Animal Density | 0.130 | 0.029 | 4.519 | >0.0001 |
| Latitude : Habitat Type (shrubland) : Aninal Density | -0.002 | 0.003 | -0.648 | 0.5171 |
| Latitude : Habitat Type (grassland) : Animal Density | -0.003 | 0.001 | -4.358 | >0.0001 |

**Figure S4:** Relationship between animal density and latitude.


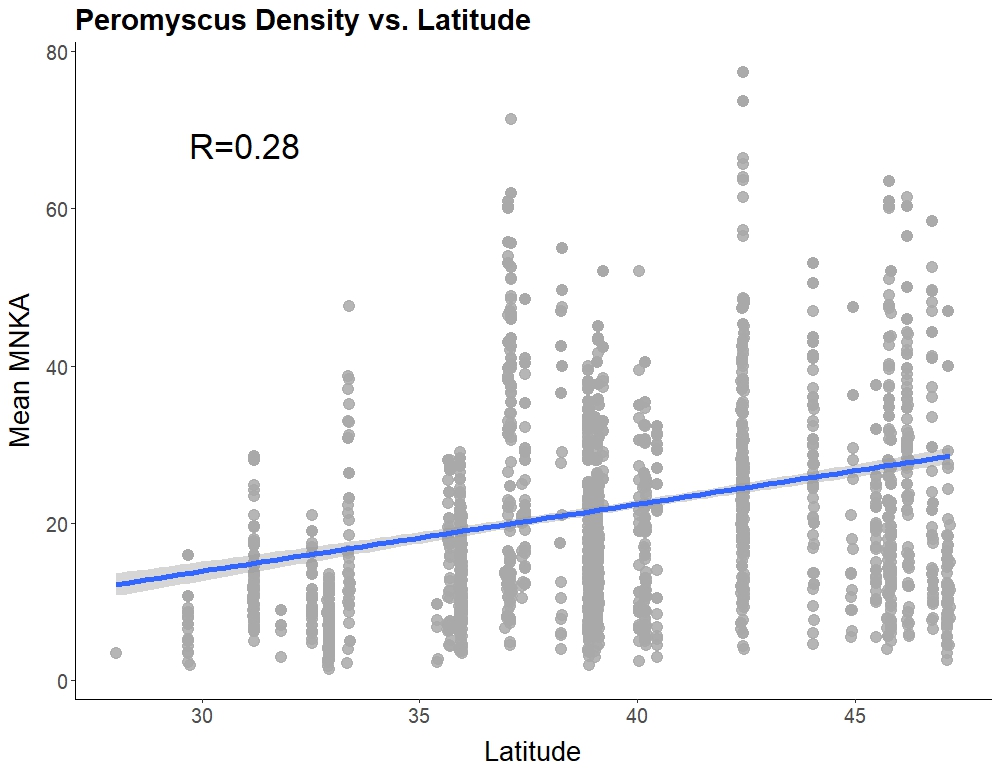


**Table S3:** Number of individuals from each *Peromyscus* species (columns) at each NEON site (rows) in our dataset. Note that the most abundant species (PEMA and PELE) occur at almost all NEON sites.


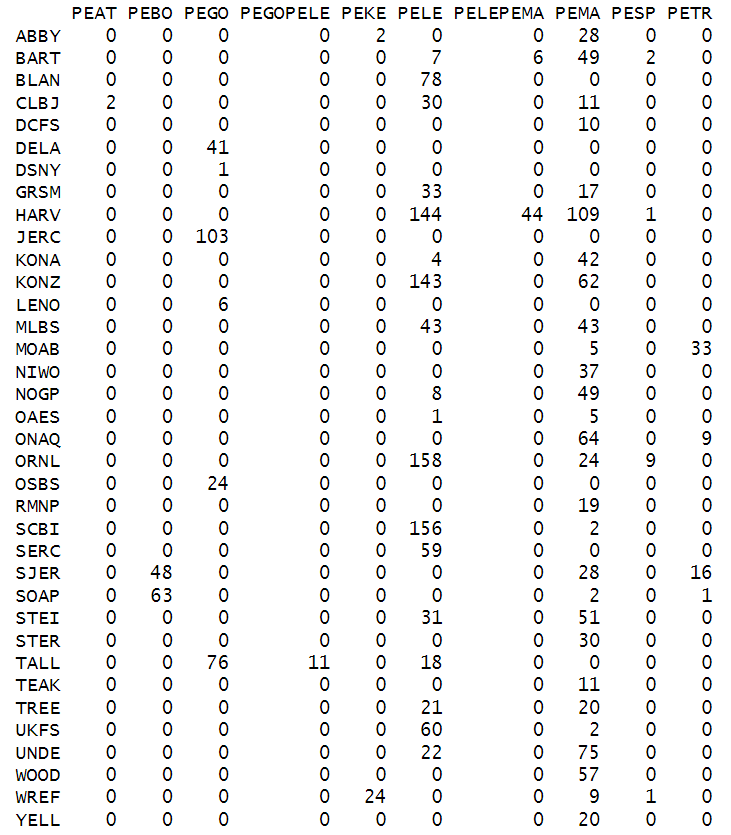


**4. Map of NEON field sites used in analysis**

**Figure S5:** Map indicating the location of each NEON field site used in this analysis.

**
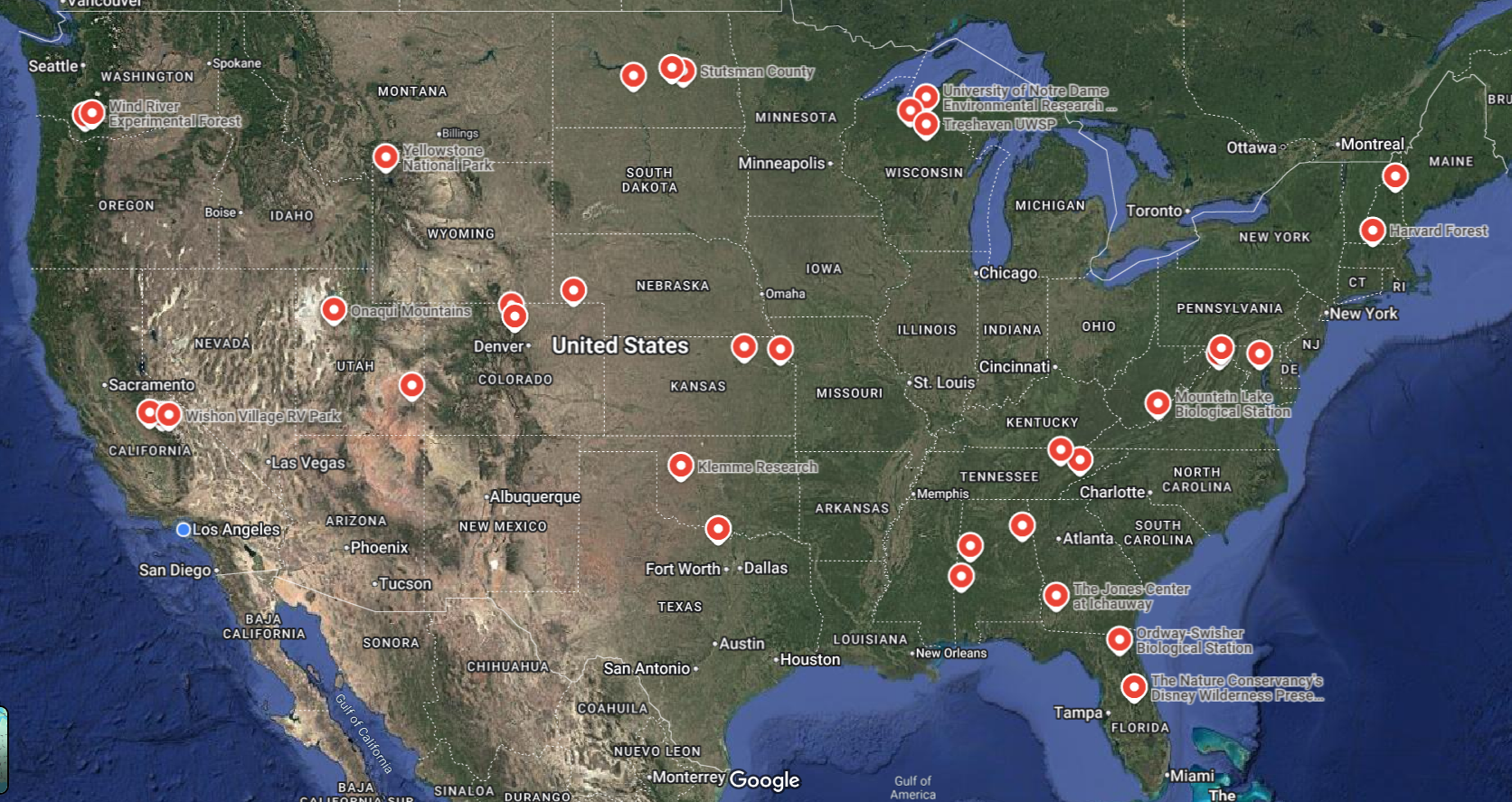
**
